# Extending the shelf life of ⁶⁸Ge/⁶⁸Ga generators via preconcentration of [⁶⁸Ga]GaCl₃ for preclinical application

**DOI:** 10.1186/s41181-025-00406-y

**Published:** 2025-11-26

**Authors:** Hemantha Mallapura, Olof Eriksson

**Affiliations:** Science For Life Laboratory, Department of Medicinal Chemistry, Uppsala University, Uppsala, Sweden

**Keywords:** ^68^Ga, Generator shelf-life, Preconcentration, Strong cation exchange, Radiolabeling, PET tracers, Affibody, Apparent molar activity

## Abstract

**Background:**

⁶⁸Ga-labeled tracers are increasingly important for PET imaging and as companion diagnostics with new therapeutic radiopharmaceuticals. The ⁶⁸Ge/⁶⁸Ga generator is a common source for clinical and preclinical ⁶⁸Ga-tracer production; however, its shelf life and efficiency are critical because of the high cost of generator replacement. This study assessed whether preconcentration of [⁶⁸Ga]GaCl₃ using strong cation exchange (SCX) resin can increase yield, apparent molar activity (AMA) and also extend generator shelf-life for preclinical applications.

**Results:**

A ⁶⁸Ge/⁶⁸Ga generator (1.8 GBq at purchase, 14–18 months old) was used. Direct elution involved elution of [⁶⁸Ga]GaCl₃ in 0.1 M HCl, followed by addition to a DOTA-conjugated affibody in acetate buffer (pH 4.6). Preconcentration involved trapping [⁶⁸Ga]GaCl₃ on a SCX (Chromafix PS-H⁺) cartridge, rinsing, and eluting with 0.12 M HCl in 5 M NaCl (300–500 µL), followed by radiolabeling. Radiolabeling was performed at 75–80 °C for 15 min, the products were purified using a NAP-5 column, and the purity was assessed by high-performance liquid chromatography (HPLC). The SCX cartridge trapping efficiency was > 99%, with a elution efficiency of 95.2 ± 1.2% (n = 8). For direct elution, the decay-corrected radiochemical yield (RCYdc) was 78.7 ± 1.5% (n = 3), the AMA was 5.6 ± 0.4 MBq/nmol, and the radiochemical purity (RCP) was 95.3 ± 0.6%. For preconcentration, RCY_dc_ was 69.0 ± 10.0% (n = 3), AMA was 12.6 ± 2.1 MBq/nmol, and RCP was 95.7 ± 3.0%.

**Conclusions:**

The preconcentration technique doubled the product yield and AMA, and extended the shelf life of the generator by 9–12 months for preclinical applications. Preconcentration of [⁶⁸Ga]GaCl₃ using SCX resin is a robust, cost-effective method for maximizing ⁶⁸Ga recovery and increasing the radiotracer yield and AMA, especially with older generators. This approach extends generator shelf-life, supports sustained preclinical research, and reduces radioactive waste and operational costs.

**Supplementary Information:**

The online version contains supplementary material available at 10.1186/s41181-025-00406-y.

## Background

Gallium-68 (^68^Ga) has become a cornerstone radionuclide in positron emission tomography (PET) because of its favorable decay properties, generator-based availability, and expanding clinical and preclinical applications of ^68^Ga-labeled radiopharmaceuticals. The clinical success of tracers such as [^68^Ga]Ga-DOTA-TOC, [^68^Ga]Ga-HBED-PSMA, and [^68^Ga]Ga-DOTA-FAPI analogs demonstrates the versatility of ^68^Ga for imaging a variety of malignancies (Syed et al. 2020; Kallur et al. 2017; Breeman et al. 2005), and the development of new chelators and targeting ligands continues to broaden its utility (Davey and Paterson 2022). The widespread adoption of ^68^Ga radiopharmacy is underpinned by the ^68^Ge/^68^ Ga generator, which provides a cyclotron-independent, on-demand source of radioactivity suitable for both small- and large-scale production (Romero et al. 2020). Recent reviews highlight the rapid evolution of ^68^Ga-tracers development, including advances in chemistry, automation, and regulatory frameworks, which have facilitated clinical translation and innovation in precision oncology (Kleynhans et al. 2024).

However, the utility of the ^68^Ge/^68^Ga generator is tempered by a gradual decline in elution yield and increasing metallic impurities as the generator ages, which can adversely affect radiolabeling efficiency, apparent molar activity (AMA), and eventually, the quantity and quality of the PET tracer produced. Conventional direct elution methods, which involve collecting [^68^Ga]GaCl₃ in low-molar hydrochloric acid and using it immediately for labeling (Breeman et al. 2011) are simple and widely used; however, as generator performance decreases, these methods often yield suboptimal product amounts and AMAs, particularly for high-demand preclinical or translational research because of ^68^Ge decay.

To overcome these limitations, preconcentration methods using strong cation exchange (SCX) resins have been developed (Mueller et al. 2016, 2012). These techniques allow quantitative trapping and subsequent elution of ^68^Ga in a smaller volume, thereby increasing the concentration of radioactivity and improving product activity and AMA even with older generators (Mueller et al. 2012; Schultz et al. 2013). SCX-based preconcentration has demonstrated the ability to extend generator shelf-life, increase tracer amount, and support cost-effective preclinical studies by maximizing residual activity (Mueller et al. 2016). Despite these advances, there remains a need for comprehensive, systematic comparisons between direct elution and SCX-based preconcentration, especially in the context of extending generator utility in sustained preclinical research.

The present study aims to address these gaps by systematically assessing the impact of SCX preconcentration on [^68^Ga]GaCl₃ recovery, radiolabeling yield, and AMA by using a DOTA-conjugated affibody as a model system. We specifically evaluate the performance of both direct elution and preconcentration protocols with 14–18 months old ^68^Ge/^68^Ga generator and discuss the implications for extending generator shelf-life, optimizing resource utilization, and supporting sustained preclinical research.

## Methods

### Materials

All radiolabeling experiments were conducted using a ⁶⁸Ge/⁶⁸Ga generator (GalliaPharm®, Eckert & Ziegler, IGG100-50 M-NT, Germany), which was 14–18 months old during the experiments. Elution of the generator was performed using ultra pure 0.1 M HCl in ultrapure water. In short 0.1 M HCl solution was prepared by diluting 1.0 mL of ultrapure hydrochloric acid (32–35%, 1.17 g/mL; VWR NORMATOM, Art. No. 83878.230), to a final volume of 100 mL ultratracer elemental analysis grade water (Fisher Scientific UK, Art. No. W9-1). For the preconcentration protocol, strong cation exchange (SCX) cartridges (Chromafix PS-H⁺, Macherey–Nagel GmbH & Co., Art. No. 731867) were used. DOTA-conjugated affibody molecules (DOTA-Cys-ATH001 also referred to as DOTA-Z09591 in the literature) were sourced from Almac, Scotland.

Acetate buffers were used as specified: pH 4.6 buffer (Honeywell Fluka, Art. No. 31048, 500 mL, Germany) and 1 M pH 5.5 buffer (Thermo Scientific, Art. No. J61033, 250 mL, Germany). Sodium chloride solution (5 M, analytical standard; Fisher Chemicals, Art. No. 12953324) was used to prepare 0.12 M HCl in 5 M sodium chloride for SCX cartridge elution, and phosphate-buffered saline (PBS) tablets (Merck, Art. No. 524650–1, Darmstadt, Germany) were used to prepare PBS for the final product formulation. Product purification was performed via NAP-5 Sephadex G-25 DNA-grade columns (Cytiva, Art. No. 17085302, Uppsala, Sweden). Reactions were carried out in 1.5 mL Eppendorf LoBind Tubes (Eppendorf, Art. No. 022431081, Hamburg, Germany). pH was monitored via DOsatest pH test strips (VWR Chemicals, pH 0.0 to 6.0).

Radioactivity measurements were conducted via a Capintec CRC 15R dose calibrator. Analytical HPLC was carried out on a VWR HITACHI Chromaster system equipped with a TOSOH BIOSCIENCE analytical column (Art. No. 0022831; 2.0 mm ID × 10 cm L, 3.0 µm particle size). Chromatographic separations were achieved via HPLC-grade acetonitrile (Supelco, Art. No. 1.00030.2500, Merck, USA) and in-house 18 MOhm water, both containing 0.1% trifluoroacetic acid (TFA, > 99%; Sigma Aldrich, Art. No. 302031, 100 mL, France).

### Preparation of [^68^Ga]GaCl₃

*Direct elution:* The ^68^Ge/^68^ Ga generator was eluted with 5 mL of 0.1 M hydrochloric acid (metal free). The fraction containing the highest ^68^Ga concentration (2.0 to 2.5 mL) was collected in a lead shielded, Eppendorf tube and used for all subsequent labeling steps.

**Fig. 1:**
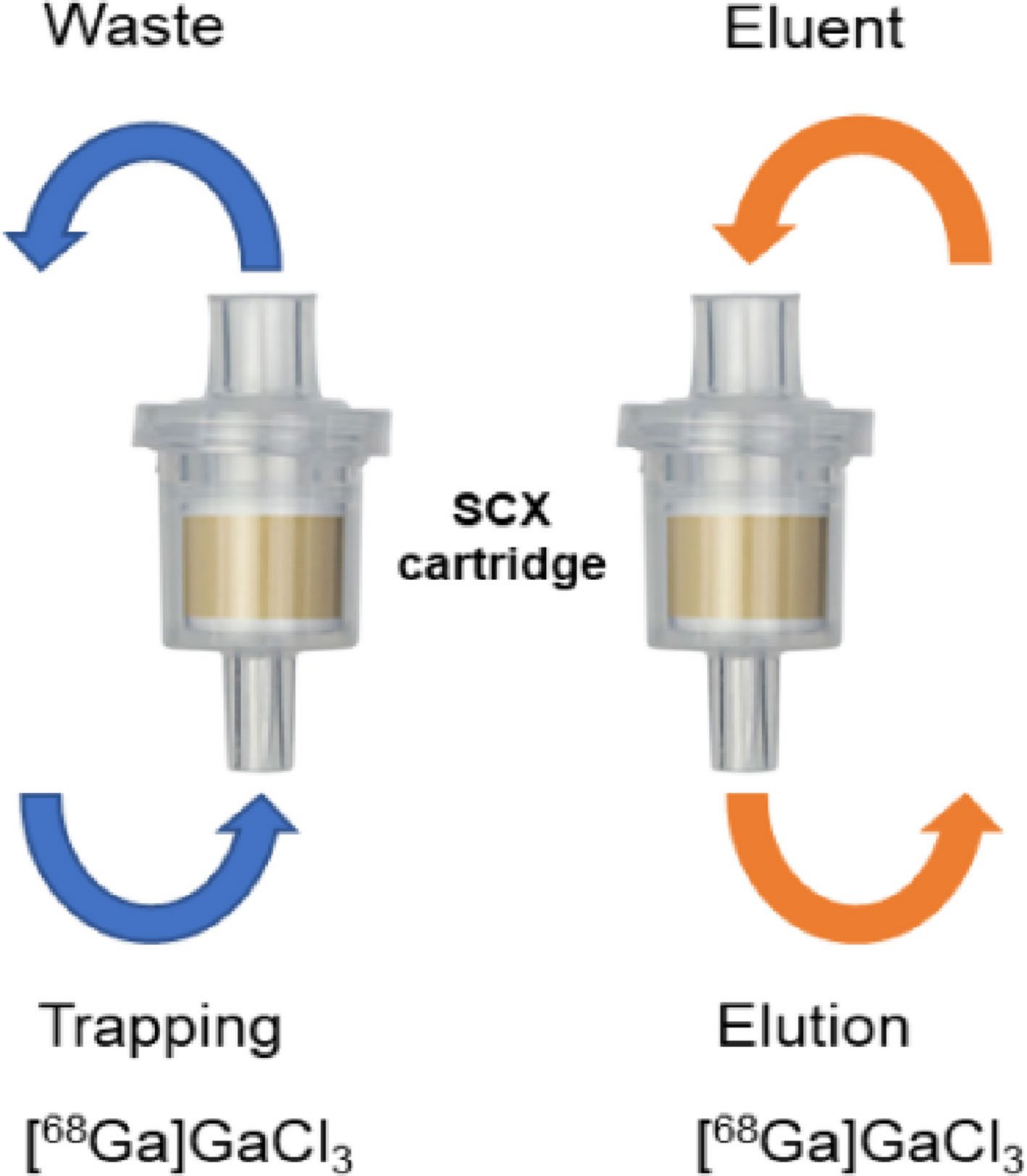
Schematic representation of the SCX cartridge showing the forward flow (blue arrows) applied during trapping and the reverse flow (orange arrows) applied during elution of [^68^Ga]GaCl_3_

*Preconcentration protocol*: The SCX cartridge (Chromafix PS-H⁺) was preconditioned with 2 mL of 0.1 M HCl, identical to the solution used for generator elution. For the SCX preconcentration protocol, the entire 5 mL generator eluate was passed slowly through a preconditioned SCX cartridge at room temperature. [^68^Ga]GaCl₃ was trapped in one direction and eluted in opposite direction to increase elution efficiency and illustration of pre-concentration shown in the Fig. 1. The cartridge was then washed with 1 mL of ultratracer-grade water to remove residual hydrochloric acid, followed by flushing with an empty syringe three times. Finally, the trapped activity was slowly eluted using 300—500 µL of 0.12 M HCl in 5 M NaCl. The trapping and elution were performed in the reverse direction.

### Radiolabeling of DOTA-conjugated affibody

**Fig. 2:**
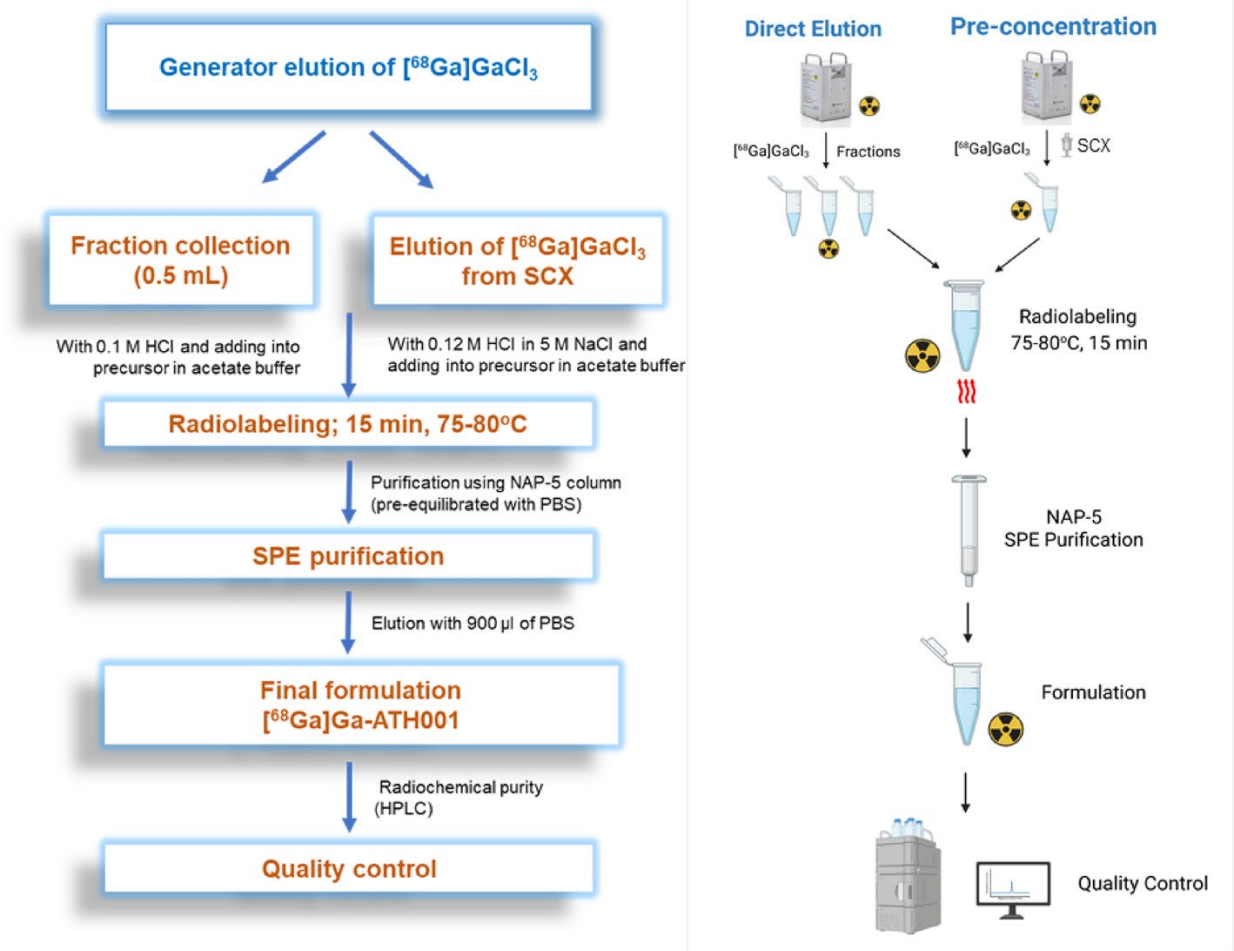
Schematic workflow and illustration for the radiolabeling of [^68^Ga]Ga- ATH001 using direct elution and pre-concentration protocols. The flowchart (left) and schematic (right) illustrate the stepwise process from generator elution, through either direct fraction collection or pre-concentration via SCX cartridge, followed by radiolabeling, solid-phase extraction (SPE) purification, final formulation, and quality control

Schematic workflow (left) and illustration (right) for the radiolabeling of [^68^Ga]Ga-DOTA-ATH001 using direct elution and pre-concentration protocols showed in the Fig. 2.

*Direct elution (n* = *3)*: 200 µg of DOTA-ATH001 (Strand et al. 2014) affibody was dissolved in 200 µL of acetate buffer (pH 4.6) to prepare a stock solution. An aliquot (40–50 µg) was transferred to a 1.5 mL LoBind Eppendorf tube, and 170 µL of acetate buffer (pH 4.6) was added. Next, 300 µL of freshly eluted [⁶⁸Ga]GaCl₃ was added. The tube was sealed with 500 µl of reaction volume and incubated at 75–80 °C for 15 min. The mixture was cooled to room temperature.

*SCX preconcentration (n* = *3)*: [⁶⁸Ga]GaCl₃ was eluted from the SCX cartridge with 300–500 µL of 0.12 M HCl in 5 M NaCl into a 1.5 mL Eppendorf tube. From this eluate, 225 µL was transferred to a 1.5 mL LoBind Eppendorf tube containing 50–70 µg of affibody stock solution and 225 µL of acetate buffer (pH 5.5), giving a final reaction volume of 500 µL. The tube was sealed and incubated at 75–80 °C for 15 min. The mixture was cooled to room temperature.

For both, the cooled reaction mixture was loaded onto a NAP-5 column (Sephadex G-25 DNA grade) preconditioned with PBS. The radiolabeled product was eluted with 900 µl of PBS. The RCP of the final product was determined using analytical high-performance liquid chromatography (HPLC). Chromatographic separation was performed over 10 min via a linear gradient from 10 to 90% acetonitrile and 90% to 10% 18 MOhm water, both of which contained 0.1% trifluoroacetic acid. The flow rate was 1 mL/min, and detection was carried out at a UV wavelength of 220 nm. Preparations exhibiting an RCP of 90% or higher were considered suitable for further studies.

### Data analysis

The following parameters were calculated and used to compare the direct elution and SCX preconcentration methods:

- Trapping efficiency (TE): The proportion (%) of total ^68^Ga activity from the generator eluate retained on the SCX cartridge.
- Elution efficiency (EE): The proportion (%) of trapped ^68^Ga activity released from the SCX cartridge during elution.
- Radiochemical yield: The percentage of total starting ^68^Ga incorporated into the purified product, decay-corrected to the start-of-synthesis (SOS).
- Apparent molar activity (AMA): The radioactivity of the purified product per nanomole of affibody (MBq/nmol), calculated at EOS.

All measurements, including trapping efficiency (TE), elution efficiency (EE), decay-corrected radiochemical yield (RCY_dc_), and apparent molar activity (AMA), were performed in triplicate unless stated otherwise. The data are presented as the means ± standard deviations (SDs), unless otherwise specified. All the graphs and statistical analysis presented here were generated via GraphPad Prism 10.4.2 (633).

## Results

### Generator performance

Radiolabeling experiments were performed using a GalliaPharm ^68^Ge/^68^Ga generator that was 14 months old at the time of start and 18 months old at the end of the study. The generator’s total elutable activity in 5 mL of 0.1 M HCl (ultrapure) decreased from 1400 MBq at purchase to 470 MBq at 14 months.

### SCX preconcentration

The preconcentration step took approximately 5 min. The SCX resin demonstrated excellent trapping efficiency (TE), consistently exceeding 99% (n = 19) across all the experiments and details of the experiments result are provided in Tables S1 and S2 (supplementary data). Elution efficiency (EE), averaged 95.2 ± 1.1% (n = 8), 94.4 ± 2.9% (n = 4), and 92.4 ± 1.9% (n = 7) when 500 µL, 400 µL, and 300 µL of 0.12 M HCl in 5 M NaCl were used as the eluent, respectively. These results confirm the robustness and reproducibility of the SCX protocol for the quantitative preconcentration of ⁶⁸Ga from generator eluate even when as little as 300 µL of eluent is used**.**

### Radiolabeling outcome

**Fig. 3:**
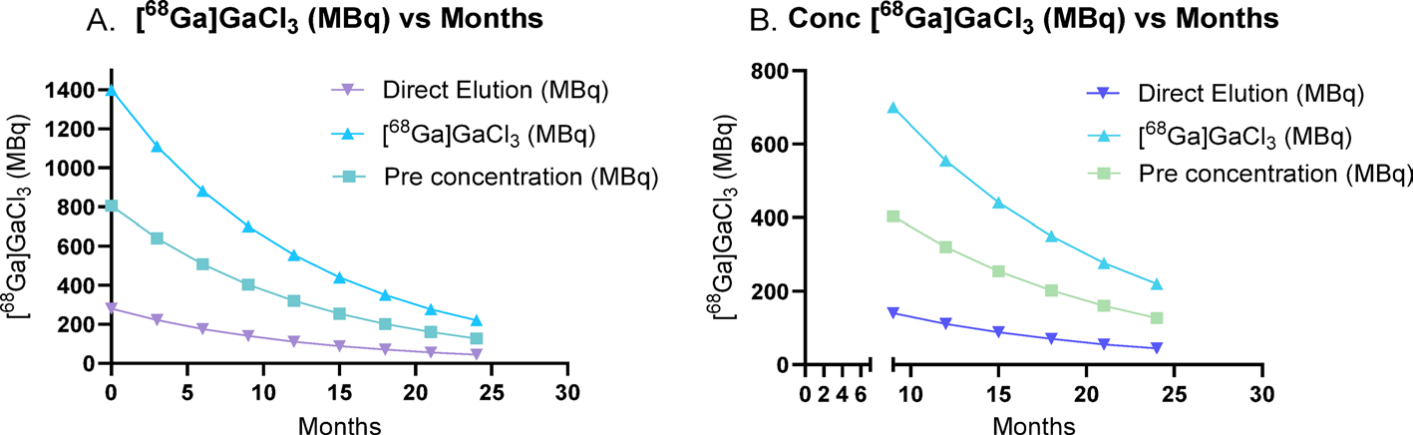
Comparision of usable ^68^Ga from the direct elution and preconcentration of ^68^Ge/^68^Ga generator elution profile over 24 months. **A** – 300 µL used from the 2.0—2.5 mL fraction in the total of 5 mL. **B** – 225 µL used from the 300 µL of ^68^Ga-eluate (0.12 M HCl in 5 M NaCl) and recovery from SCX more than 90%

Radiolabeling of the DOTA-conjugated affibody (DOTA-ATH001) was performed using [⁶⁸Ga]GaCl₃ obtained from both direct elution and SCX-based preconcentration methods. The results are summarized in Table 1. For the direct elution method, the recovery of [⁶⁸Ga]GaCl₃ in 500 µL was approximately 30% (Fig. 3), resulting in a final product activity of 39.7 ± 3.1 MBq. The RCY_dc_ was 78.7 ± 1.5%, the RCP was 95.3 ± 0.6%, and the AMA was 5.6 ± 0.4 MBq/nmol. In contrast, the preconcentration protocol achieved significantly higher recoveries of [⁶⁸Ga]GaCl₃: 95.2 ± 1.1% (Fig. 3) using 500 µL, 94.4 ± 2.9% using 400 µL, and 92.4 ± 1.9% (p < 0.05) using 300 µL of eluent, yielding a product activity of 99.7 ± 24.9 MBq (p < 0.05). The RCY_dc_ was 69.0 ± 10.0%, with an RCP of 95.7 ± 3.1%, and notably higher AMA of 12.6 ± 2.2 MBq/nmol (*p* < 0.01).

**Table 1:** Radiolabeling data for both direct elution and preconcentration methods

Elution type	Recovery of [^68^ Ga]GaCl_3_ in 500 µL (%)	Starting activity (MBq)	Precursor concentration (nmol)	Product (MBq)	Yield_dc_ (%)	RCP (%)	Molar activity (MBq/nmol)
Direct (n = 3)	30	64 ± 5	6.25	39.7 ± 3.1	78.7 ± 1.5	95.3 ± 0.6	5.6 ± 0.4
Preconcentration (n = 3)	95	221 ± 14*	8.75	99.7 ± 24.9*	69.0 ± 10.0	95.7 ± 3.05	12.6 ± 2.1**

*represents *p* < 0.05, and **represents *p* < 0.01 compared to the direct elution method

Both methods consistently produced radiolabeled affibody products with high radiochemical purity (Direct—95.3 ± 0.6 and Preconcentration—95.7 ± 3.05 (n = 3 for both)), which are suitable for preclinical applications (Supplementary data Figure S1). The formulations remained stable in PBS for more than 1 h, retaining > 90% RCP. The total synthesis time for the direct elution method was approximately 28 min, whereas the inclusion of the preconcentration step increased the total time by only 5 min.

## Discussion

The present study demonstrates the practical value of applying SCX-based pre-concentration to extend the useful operational lifespan of aging ^68^Ge/^68^Ga generators in a preclinical setting. While the ability of pre-concentration to increase the amount of usable [^68^Ga]GaCl_3_ is well established, the primary focus of this work was not to revalidate this known principle. Instead, our aim was to systematically assess its applicability under real-world conditions using an 18-month-old generator, where elution yield and apparent molar activity typically decline to levels that hinder routine radiolabeling. Our findings show that SCX pre-concentration effectively compensates for this age-related decline, enabling consistently high product and radiochemical purity and adequate molar activity even at later stages of generator life. This is particularly relevant for research laboratories and small-scale facilities that depend on a single generator for extended use, where frequent generator replacement poses substantial financial and logistical challenges. Thus, the strength of this study lies in demonstrating the continued utility, sustainability, and cost-effectiveness of a well-established technique under long-term operational conditions. Overall, this study results support broader adoption of SCX pre-concentration as a green, economically viable, and resource-efficient strategy for maximizing generator performance and reducing waste across both preclinical and translational research environments.

### Direct elution and preconcentration

The longitudinal evaluation of the ⁶⁸Ge/⁶⁸Ga generator over a two-year period highlights the practical and economic benefits of incorporating a preconcentration protocol for [⁶⁸Ga]GaCl₃ recovery. The data of usable ^68^Ga from direct and preconcentration elution from the generator are shown in Fig. 3 (supplementary data Table S3). During the first 9–12 months of generator use, direct elution alone yielded sufficiently high activity (e.g., Fig. 3.A.; 280 MBq at startup and 110 MBq at 12 months) to meet the requirements of most preclinical applications. Under these conditions, the simplicity and speed of direct elution make it the preferred method, as the recovered activity is adequate for routine radiolabeling and preclinical imaging studies.

However, as the generator ages and its output declines, the activity obtained by direct elution alone eventually falls below the threshold required for effective preclinical or translational research. Beyond 12 months, the yield from direct elution decreases substantially (e.g., < 50 MBq at 24 months), often rendering the generator unsuitable for further use by conventional means. In contrast, the implementation of a preconcentration protocol at this stage significantly increases the recovery and concentration of ^68^Ga (e.g., Fig. 3.B.; 320 MBq at 12 months and > 120 MBq at 24 months in 300 µL), thereby extending the operational life of the generator for up to an additional 12 months.

This extension is not only technically advantageous, allowing the continued production of high-activity in small-volume eluates suitable for demanding radiolabeling methods but also economically beneficial. By maximizing the extraction of usable ^68^Ga from each generator, preconcentration reduces the frequency of generator replacement and the associated costs of procurement, installation, and regulatory compliance. This approach supports more sustainable and cost-effective preclinical research, particularly in settings where budget constraints or supply chain limitations may impact the availability of new generators.

### Preconcentration performance

The SCX preconcentration protocol demonstrated outstanding performance in both the trapping and elution of [⁶⁸Ga]GaCl₃, as summarized in Table 1. Across 19 independent experiments, the trapping efficiency was remarkably high at 99.8 ± 0.3%, emphasizing the reliability and reproducibility of the SCX cartridge for ^68^Ga preconcentration. With respect to eluting the trapped ⁶⁸Ga, the protocol also proved highly effective. Using 500 µL of 0.12 M HCl in 5 M NaCl, the elution efficiency averaged 95.2 ± 1.2% (*n* = 8), indicating that almost all of the trapped radioactivity could be recovered in a small, concentrated volume. Even when the elution volume was reduced to 400 µL and 300 µL, the efficiency remained high at 94.4 ± 2.9% (*n* = 4) and 92.4 ± 1.9% (*n* = 7), respectively. The statistical analysis (one-way ANOVA) showed a significant difference in elution efficiency (EE) between 500 µL and 300 µL. However, this difference is not practically meaningful, as the activity concentration increased by approximately 1.5-fold with only a 3% loss of the total activity.

The comparative data in Table 1 clearly demonstrate the substantial advantage of SCX preconcentration over direct elution, particularly as the generator ages. For example, with a 15-month-old generator, direct elution yielded only 64 ± 5 MBq (n = 3), whereas preconcentration enabled recovery of 221 ± 14 MBq (n = 3), nearly three times greater. Notably, this recovered activity using preconcentration at 15 months is equivalent to what would be obtained from direct elution with a generator just 3 months of age (222 MBq; Fig. 3.A and B). This finding indicates that preconcentration not only offsets the natural decrease in generator output but also essentially restores the available activity to levels comparable to those of a newer generator. This substantial enhancement in recovery enables the acquisition of sufficient activity for demanding preclinical experiments, even as the generator ages, thereby extending its operational lifespan and ensuring a continuous supply of activity from the same generator for up to two years.

### Radiolabeling: direct elution and preconcentration

All the results presented in this study were obtained via a ⁶⁸Ge/⁶⁸Ga generator that had been in operation for 14–18 months. This context is critical, as it highlights the limitations of direct elution for routine preclinical applications beyond the first year of generator use. With direct elution, the lower recovery of [⁶⁸Ga]GaCl₃ and reduced product activity may limit studies that require higher injected doses or imaging of multiple animals. The preconcentration method effectively addresses these limitations by enabling the recovery of substantially more radioactivity in a small volume, thereby increasing both the product activity and apparent molar activity (AMAs). This is especially beneficial for studies of low-density receptors, where high AMA is essential to minimize tracer mass, avoid receptor saturation, and maintain biological specificity. In such scenarios, preconcentration can be selectively applied to maximize tracer performance, ensuring that even with an aging generator, high-molar-activity products remain available for demanding preclinical research.

Although both direct elution and preconcentration protocols employ similar radiolabeling conditions -incubation at 75–80 °C for 15 min, there are notable differences in the reaction environment due to variations in the composition and concentration of the labeling solutions. In the direct elution method, acetate buffer at pH 4.6 was used to maintain the optimal reaction pH. In contrast, the preconcentration method delivers [⁶⁸Ga]GaCl₃ in a significantly smaller volume of 0.12 M HCl in 5 M NaCl, resulting in a slightly lower starting pH than that of standard 0.1 M HCl. To compensate, a higher-strength acetate buffer (1 M, pH 5.5) was used in the preconcentration protocol to achieve a suitable reaction pH and ensure radiolabeling yields exceeds more than 80%.

For direct elution, a precursor concentration of 6.25 nmol was used, while preconcentration required a slightly greater amount of 8.75 nmol. When 6.25 nmol of precursor was used with the preconcentration eluate, the radiolabeling yield was limited to 60–70% (*n* = 3), likely due to the high salt content from the 5 M NaCl. Increasing the precursor to 8.75 nmol improved the radiolabeling yield to 83 ± 12% (*n* = 3). In both protocols, the total reaction volume was standardized at 500 µL to allow compatibility with NAP-5 column purification. However, owing to the smaller elution volume and higher activity concentration, the radioactivity per milliliter was approximately three times greater with the preconcentration method.

The results summarized in Table 1 clearly demonstrate the superiority of the preconcentration protocol over direct elution for recovering and radiolabeling [⁶⁸Ga]GaCl₃ from 14–18 months ⁶⁸Ge/⁶⁸Ga generator. While direct elution yielded only 30% recovery of [⁶⁸Ga]GaCl₃ in 300 µL (2.0 to 2.5 mL fraction) from a 5 mL generator eluate, preconcentration achieved a remarkable 95% recovery in the same volume. This resulted in a final product activity of 99.7 ± 24.9 MBq (n = 3) for the preconcentration method, which was more than double that obtained with direct elution 39.7 ± 3.1 MBq (n = 3). Although the RCY_dc_ for preconcentration (69.0 ± 10.0%; n = 3) was slightly lower than that for direct elution (78.7 ± 1.5%; n = 3), this modest difference is outweighed by the significantly greater amount of usable radioactivity recovered, which enables more efficient and scalable preclinical applications.

A key benefit of the preconcentration approach is the significant increase in apparent molar activity (AMA), which was as high as 12.6 ± 2.1 MBq/nmol when an 18-month-old generator was used, whereas only 5.6 ± 0.4 MBq/nmol was observed with direct elution. Elevated AMA is essential for preclinical imaging in high-affinity receptor-targeted studies, as it permits the administration of lower peptide doses and minimizes the risk of receptor saturation or unwanted pharmacological effects. Statistical analysis (t-test) revealed a significant difference in RCY and AMA between the direct elution and pre-concentration methods. The results clearly demonstrate that the pre-concentration method is superior to direct elution, providing higher product activity and AMA. Furthermore, the preconcentration technique can be utilized with new generators when higher apparent molar activity is needed.

### Sustainability perspective and green radiochemistry

The implementation of SCX preconcentration for ^68^Ga recovery significantly advances the principles of green radiochemistry by maximizing radionuclide utilization and minimizing waste. Our data demonstrate that preconcentration achieves a recovery of ^68^Ga as high as 95% from generator eluate, compared to only about 30% recovery by direct elution (Table 1). This more than threefold increase in usable radioactivity substantially reduces the frequency at which costly ^68^Ge/^68^Ga generators must be replaced—extending the operational lifespan by up to 12 months. Such an extension directly translates to decreased radioactive waste generation, lower raw material consumption, and reduced environmental footprint associated with generator manufacturing, transport, and disposal. Furthermore, the preconcentration method yields a doubling of apparent molar activity (12.6 ± 2.1 MBq/nmol vs. 5.6 ± 0.4 MBq/nmol for direct elution), enabling more efficient radiolabeling with less precursor material. This resource efficiency is central to sustainability, reducing chemical and biological reagent consumption and minimizing potential ecological impact. Collectively, these advantages establish SCX preconcentration as a robust, cost-effective, and environmentally responsible strategy for sustainable radiopharmaceutical production.

### Economic and practical benefits

The economic impact of extending the operational lifespan of a ⁶⁸Ge/⁶⁸Ga generator by up to 12 months is substantial. Given the high cost of these generators, the ability to continue using a single unit for two full years rather than replacing it annually can lead to significant savings, particularly for academic and preclinical research programs operating under tight budgets. For example, a laboratory that typically replaces its generator every year to maintain a reliable ⁶⁸Ga supply could reduce generator-related costs by 50% over a two-year period simply by adopting the preconcentration method. From a practical standpoint, the protocol is easy to implement, requiring only an additional five minutes per synthesis for the washing and elution steps. This small increase in preparation time fits seamlessly into existing workflows and is more than justified by the overall gains in efficiency and cost-effectiveness. By maximizing the recovery of usable ⁶⁸Ga, the preconcentration approach helps laboratories reduce recurring expenses, minimize disruptions caused by generator replacement, and better allocate resources toward research and development. In summary, this strategy offers a practical, efficient, and economically sound solution for extending generator utility and optimizing operational resources across both research and clinical settings, while simultaneously supporting the principles of green and sustainable radiochemistry.

### Ongoing work and future directions

While the current preconcentration protocol has proven robust and effective for extending generator shelf-life and maximizing both product yield and AMA, ongoing work is focused on further optimization on the basis of our findings.

Given the high trapping efficiency (99.8 ± 0.3%) and strong elution efficiencies achieved with 300–500 µL volumes, we are now exploring the use of alternative elution volumes with micro SCX cartridges to further concentrate [^68^Ga]GaCl₃ and potentially increase AMA even more. The results of the current work will be applied to additional affibody targets, including CD69 and other novel molecular binders. The approach will also be expanded to modified affibody formats such as ABD-fused constructs and dimeric affibodies, as well as to theranostic applications. Furthermore, the efficacy of the pre-concentration protocol will be evaluated using older generators (12–24 months) and validated with alternative generator systems, including the iThemba generator. In addition, we are working to integrate automation into the preconcentration and labeling workflow, aiming to streamline the process, reduce manual handling, and improve reproducibility. Importantly, further studies are being conducted to test different buffer solutions and radiolabeling conditions, with the goal of optimizing the reaction environment to ensure that all the preconcentrated activity can be efficiently and quantitatively utilized. These ongoing efforts are designed to make the protocol even more adaptable and efficient for a wide range of preclinical radiochemistry applications.

## Conclusions

This study demonstrated that SCX preconcentration is a practical and effective strategy for maximizing [⁶⁸Ga]GaCl₃ recovery and utility from aging ⁶⁸Ge/⁶⁸Ga generators. In addition to doubling the product activity and significantly increasing the AMA compared with direct elution, preconcentration ensures the continued supply of the required tracer amount with high AMA even after 18 months of generator use. The protocol is simple to implement, adds minimal time to the overall synthesis workflow, and does not compromise product purity or quality. Importantly, this approach offers substantial logistical and economic advantages by extending a generator’s operational lifespan by up to 12 months, potentially saving the cost of one generator every two years. The adoption of SCX preconcentration supports more cost-effective and sustainable preclinical research and serves as a model for efficient resource utilization in radiopharmaceutical laboratories. By reducing waste and maximizing radionuclide utilization, it also aligns closely with the principles of green chemistry, promoting environmentally responsible radiopharmaceutical production.

## Supplementary Information

Below is the link to the electronic supplementary material.

Supplementary Material 1

## Data Availability

The datasets used and/or analyzed during the current study are available from the corresponding author on reasonable request.

## Declarations

### Use of AI/LLM tools

Artificial intelligence tools, including large language models (LLMs) were used to assist in improving the clarity, grammar, and language of the manuscript. No AI tools were used for data generation, analysis, interpretation, or drawing scientific conclusions.
