# Supplementary material for: Extending the shelf life of ⁶⁸Ge/⁶⁸Ga generators via preconcentration of [⁶⁸Ga]GaCl₃ for preclinical application
Source: EJNMMI Radiopharm Chem. 2025 Nov 26;10:76. doi: 10.1186/s41181-025-00406-y (PMC12669415; doi:10.1186/s41181-025-00406-y)

**Supplementary Information**

**Extending the shelf life of ⁶⁸Ge/⁶⁸Ga generators via preconcentration of [⁶⁸Ga]GaCl₃ for preclinical application**

Hemantha Mallapura^1*^ and Olof Eriksson^1^

^1^ Science For Life Laboratory, Department of Medicinal Chemistry, Uppsala University, Uppsala, Sweden.

Hemantha Mallapura (H.M.)*

[hemantha.mallapura@ilk.uu.se](mailto:hemantha.mallapura@ilk.uu.se)

Olof Eriksson (O.E.)

[olof.eriksson@ilk.uu.se](mailto:olof.eriksson@ilk.uu.se)

*Corresponding author

| **Elution volume (µL)** | 500 | 400 | 300 |
| --- | --- | --- | --- |
| **Elution Efficiency (%)** | 96.7 | 92.4 | 94.8 |
|  | 95.2 | 91.3 | 90.8 |
|  | 94.7 | 96.8 | 90.8 |
|  | 94.0 | 96.9 | 92.3 |
|  | 93.8 |  | 91.5 |
|  | 96.1 |  | 95.2 |
|  | 94.5 |  | 91.1 |
|  | 96.6 |  |  |
| **Average percentage (%)** | 95.21±1.1 | 94.4±2.9 | 92.4±1.9 |

**Table S1**. Elution efficiency (EE) of [^68^Ga]GaCl₃ from the SCX cartridge at different elution volumes.

| **Trapping efficiency (TE) %** | **Elution efficiency (EE) %** | |
| --- | --- | --- |
| 99.8±0.3% (n=19) | 500 µL | 95.2±1.1% (n=8) |
|  | 400 µL | 94.4±2.9% (n=4) |
|  | 300 µL | 92.4±1.9% (n=7)* |

**Table S2.** Summary of trapping and elution efficiency of preconcentration method of [^68^Ga]GaCl_3._

(* represents p < 0.05 compared to 500µL).

| **Month** | **Generator age (months)** | **Total [^68^Ga]GaCl₃**  **(≈ MBq)** | **Direct elution (≈ MBq)^a^** | **Pre-concentration**  **( ≈ MBq)^b^** |
| --- | --- | --- | --- | --- |
| 24-Jan | 0 | 1400 | 280 | 806 |
| 24-Mar | 3 | 1111 | 222 | 640 |
| 24-Jun | 6 | 882 | 176 | 508 |
| 24-Sep | 9 | 700 | 140 | 403 |
| 24-Dec | 12 | 555 | 111 | 320 |
| 25-Mar | 15 | 441 | 88 | 254 |
| 25-Jun | 18 | 350 | 70 | 202 |
| 25-Sep | 21 | 277 | 55 | 160 |
| 25-Dec | 24 | 220 | 44 | 127 |

**Table S3.** Comparision direct and preconcentration of ^68^Ge/^68^Ga generator elution profile over 24 months.

**Figure S1:** **Representative HPLC chromatograms of DOTA-ATH001 and [^68^Ga]Ga-DOTA-ATH001.**
(Top) UV chromatogram showing the retention time of the unlabeled DOTA-ATH001 peptide.
(Bottom) Radiochromatogram of the radiolabeled product, [^68^Ga]Ga-DOTA-ATH001, with a major peak at the same retention time as the peptide and a minor peak corresponding to free ^68^Ga.


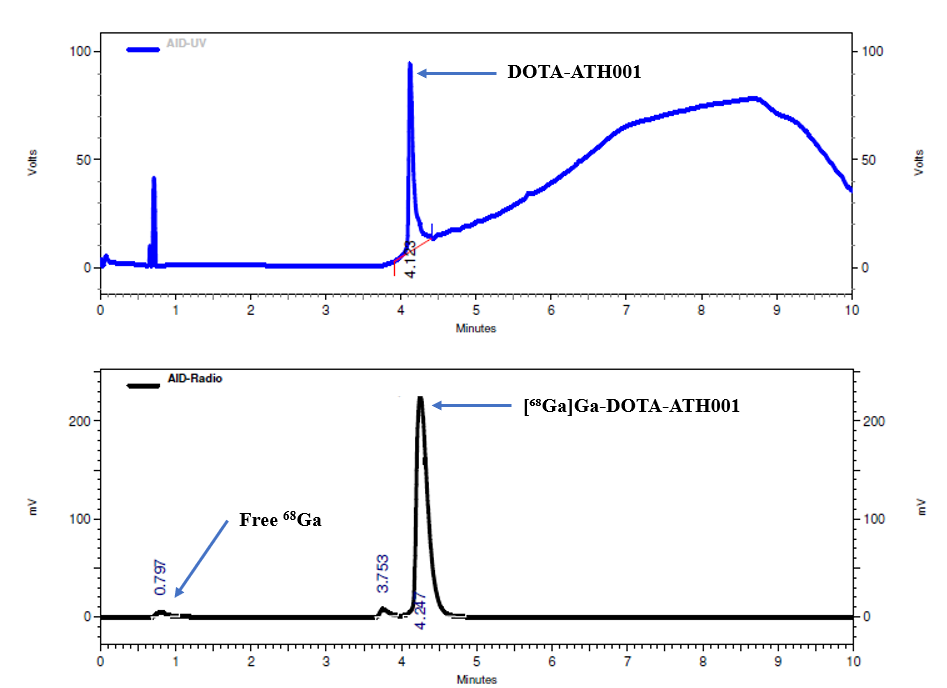

Supplement: Supplementary file 1 — Supplementary Material 1 [file 41181_2025_406_MOESM1_ESM.docx]
